# Fabrication of Pd NPs-supported porous carbon by integrating the reducing reactivity and carbon-rich network of lignin

**DOI:** 10.1038/s41598-019-43840-0

**Published:** 2019-05-13

**Authors:** Guocheng Han, Qimeng Jiang, Weijie Ye, Chuanfu Liu, Xiaoying Wang

**Affiliations:** State Key Laboratory of Pulp & Paper Engineering, South China University of Technology, Guangzhou, 510640 China

**Keywords:** Nanoparticles, Synthesis and processing

## Abstract

The renewable resource as a major feedstock to prepare porous carbon has showed many advantages compared to fossil-based materials. This study proposes a new strategy to synthesize palladium nanoparticles (Pd NPs)-supported porous carbon, utilizing both the chemical reactivity and the carbon-rich 3D network of lignin. The Pd NPs-supported porous carbons were prepared in one-pot synthesis, with Pd(NH_3_)_2_Cl_2_ as precursor, lignin as reducing and stabilizing agents of Pd NPs, nano SiO_2_ as hard-template, followed by carbonization and removal of the template. The results reveal a positive effect of Pd precursor dosage on the development and excellent texture of the Pd NPs-supported porous carbon. Accordingly, the synthesized porous carbon was proved to have large micropore volume and good micro-mesopore porous structure, revealing it a promising hydrogen adsorbent.

## Introduction

In recent years, renewable resources have gained great attention due to their abundance, renewability, biodegradability, biocompatibility and versatility compared to the less and less fossil-based resources^1,2^. It is foreseeable that a sustainable and environmentally friendly chemical industry using renewable resources as a major feedstock will displace the current fossil-based chemical industry which causes serious environmental problems^3^. Among various sustainable materials, lignin is one of the most important raw materials as it is the most abundant aromatic polymer and the second abundant biopolymer in nature, and it can be easily obtained as a waste product from the wood pulp industry^4^. With relatively high carbon content and extensively crosslinked polymeric network, lignin is considered as an excellent precursor for carbon materials such as porous carbon^5^. Therefore, notable studies have been conducted to produce porous carbons from lignin because of the aforementioned advantages^6,7^. However, there are some deficiencies limiting the industrial application of individual porous carbons, such as low specific surface area, poor pore structure and inhomogenous pore-size distribution^4,7,8^.

Recently, some studies have carried out an effective method of introducing nano noble metal in porous carbon materials to overcome these disadvantages^5,6,9^. Among them, Pd NPs- supported carbon materials have shown remarkable properties as efficient catalyst in chemical engineering processes^10^, especially in the hydrogen storage system because of the hydrogen spillover effects^11^. However, the noble metal nanoparticles are usually synthesized with toxic agents although the carbons are derived from renewable resource, which can be hardly regarded as a green protocol. Hence, it is believed to be a promising and valuable strategy if we can prepare the noble metal nanoparticles-supported carbon materials by integrating both the reducing reactivity and the carbon network of our raw material, realizing the comprehensive utilization of resources. In general, except for the 3D carbon network structures, lignin also contains easily oxidative functional groups such as hydroxyl that can act as reducing agents for the synthesis of metal nanoparticles. A study accomplished an innovative research in lignin-stabilized Pd NPs and Pt NPs, proving the possibilities of industrial residue lignin as green reducing agent in precious metal nanoparticle formation^9^. The reducing reactivity of lignin was also proved in our previous study for the green synthesis of Ag NPs, AuNPs and Au Pd alloy^3,12,13^.

**Figure 1:**
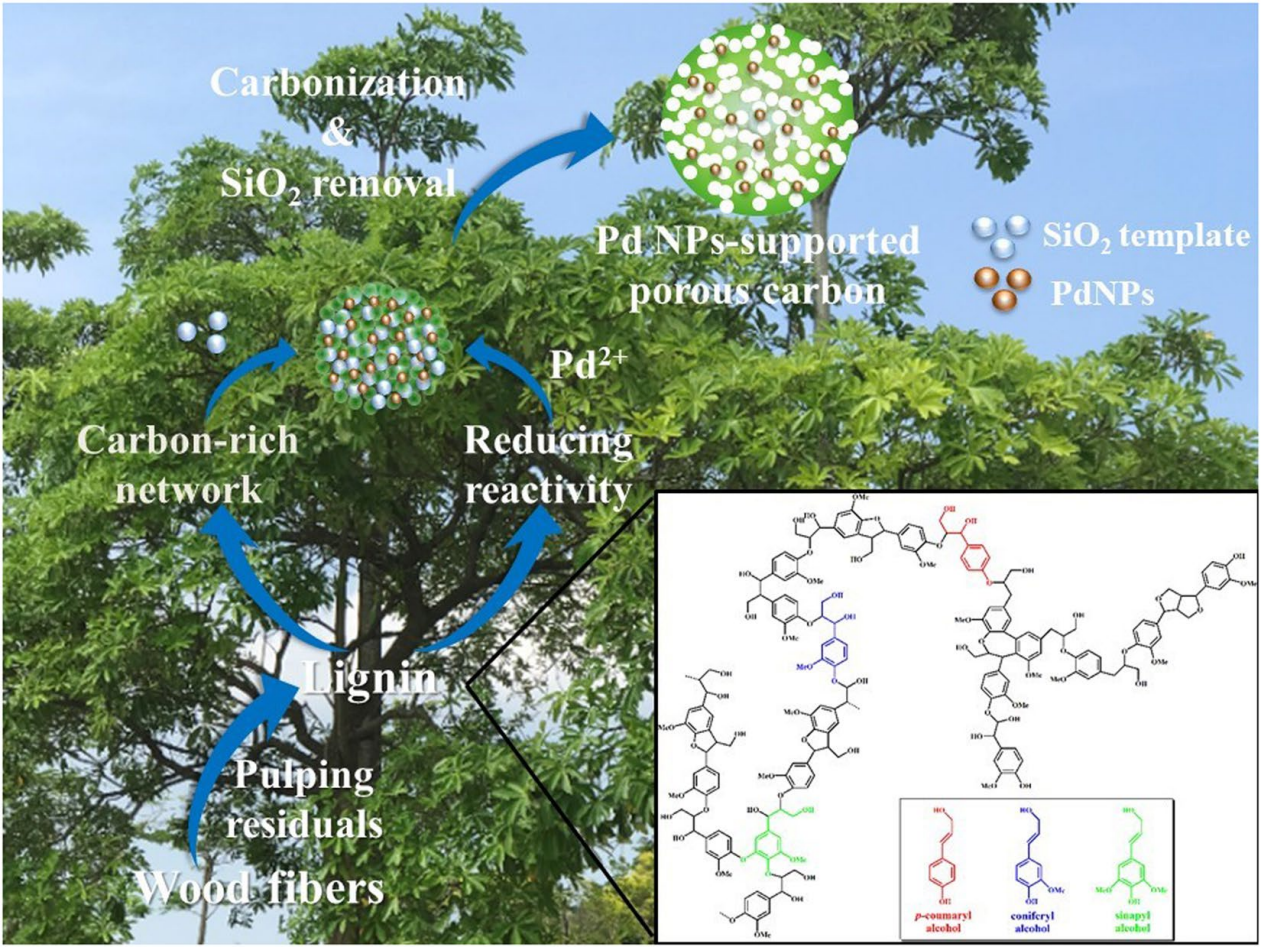
Schematic preparation of Pd NPs-supported porous carbon by combining chemical reactivity and carbon-rich network of lignin.

In this study, for the first time, in terms of making the most efficient utilization of lignin, we combined its reducing reactivity with carbon-rich network into a Pd NPs-supported porous carbon, as shown in Fig. 1. The impact of Pd NPs on the porous structure, as well as the hydrogen storage capability was investigated.

## Results and Discussion

### Green synthesis of Pd NPs with lignin

**Figure 2:**
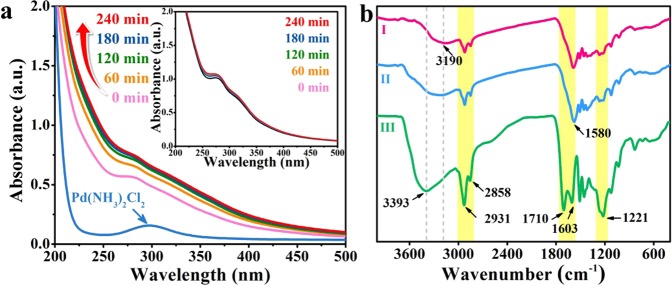
(**a**) Time-resolved UV-Vis spectra during the reduction of Pd(NH_3_)_2_Cl_2_ in lignin solution, (the inset shows the spectra of pure lignin during microwave irradiation); (**b**) FT-IR spectra of (I) lignin after 4 hours’ reduction, (II) lignin after 1 hour’s reduction and (III) original lignin.

The synthesis process was investigated by UV-Vis spectrometry, as shown in Fig. 2(a). As Pd NPs do not show pronounced surface plasmon peaks due to *d*-*d* interband transitions, the reduction of palladium salt can be distinguished by monitoring the disappearance of the diagnostic absorption at 295 nm. However, when there is lignin in the system, the strong adsorption of phenolic units within lignin at 280 nm will completely cover the above Pd(II) band throughout the reaction. Thus, the formation of Pd NPs was followed by the increasing intense of absorbance in the UV region, which is probably related to the increasing amount of Pd NPs formed. With the prolongation of reaction time, higher content of Pd nanoparticles was obtained and Pd nanoparticles provided more places for the interaction with produced carbon atoms^14^. The formation of this interaction resulted in the increased absorption of UV-Vis intensity. It can be observed that the absorbance of spectrum increased with prolonged time and changed little after 180 minutes’ reaction. Noteworthily, the absorbance of lignin solution without metal salt showed little increment during the 4 hours of time (see the inset of Fig. 2(a)), giving a possible evidence of the formation of Pd NPs.

The structural change of lignin during the reducing process was illustrated by FT-IR spectra in Fig. 2(b). Compared to original lignin (spectrum III), the band at 3393 cm^−1^ ascribed to O−H stretching of lignin after reduction (spectrum I and II) was weakened, as well as the absorption band of –CH_2_− and –CH_3_ stretching assigned to lignin structure at 2800 cm^−1^ to 3000 cm^−1^, indicating the reduction of hydroxyl within lignin, and some cut offs of lignin structure. In the spectrum of raw lignin, there was a 1710 cm^−1^ band belonged to the unconjugated C=O stretching of ester. While after the reaction, the unconjugated C=O units of ester were probably transformed to carboxylic anion (COO^−^), resulting in the disappearance of 1710 cm^−1^ band, and the occurrence of 1580 cm^−1^ band. The band around 1221 cm^−1^ corresponded to the phenolic hydroxyl stretching of lignin was diminished after the reduction, implying that some phenolic hydroxyl units of lignin may be reacted during Pd NPs formation. Furthermore, compare spectrum I with II, it can be seen with increasing reaction time, the hydroxyl stretching band decreased and shifted to lower wavenumber, which may be a result of interaction between hydroxyl of lignin and the synthesized Pd NPs.

### Structure characterization of Pd nanoparticles-supported porous carbon (PPC)

**Figure 3:**
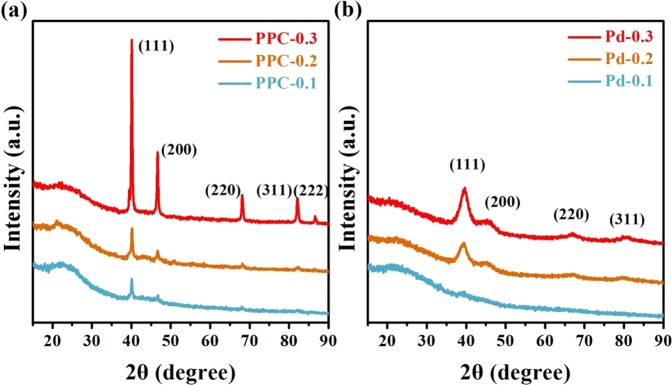
XRD patterns of (**a**) PPC with different Pd contents and (**b**) samples prepared without SiO_2_ with different Pd contents.

The XRD patterns in Fig. 3 show that the characteristic reflection peaks of face-centered cubic structure of metallic Pd (JCPDS, No. 05-0681) of PPC samples are stronger than those of non-carbonized lignin-Pd NPs composites. Besides four peaks at 2*θ* = 40.1°, 46.6°, 68.1° and 82.0° corresponding to the (111), (200), (220) and (311) facets of PdNPs^15,16^, there was one more peak at 2*θ* = 87.2° ascribed to (222) facet for the PPC-0.3 sample, as shown in Fig. 3(a). The residual Pd content detected by ICPMS is shown in Table 1, it can find that the Pd contents in Pd NPs-supported lignin-based porous carbon are higher than those of lignin-Pd composite, indicating that the reducing effects of lignin still remained in the one-pot synthesis, and the carbonization process also induced the crystal of Pd. Contrast to the Pd contents of samples prepared with SiO_2_, the samples prepared without SiO_2_ have relatively small amount of real Pd content. It indicates that the SiO_2_ as a pore-making agent could produce more pores and adsorb more Pd nanoparticles. As we all know, the shapes of peaks are basically dependent on the particle size of samples. Therefore, sharpness of the peaks in Fig. 3(a) means the size distributions of PPC samples are more uniform and concordant than those of non-carbonized lignin-Pd NPs composites in Fig. 3(b).

**Table 1:** Textural Properties of the PPC samples.

Samples	S_BET_ (m^2^/g)	V_total_^a^ (cm^3^/g)	V_micro_^b^ (cm^3^/g)	V_meso_^c^ (cm^3^/g)	Pore size^d^ (nm)	Residual Pd contents (g/g)
PPC-0.1	1028	0.89	0.08	0.81	15.60/29.80	0.4277
PPC-0.2	1128	1.03	0.06	0.97	15.70/31.60	0.5560
PPC-0.3	1467	1.34	0.18	1.16	15.80/33.40	0.6672
LPC	893	1.49	0.05	1.44	19.73	—

^a^Total pore volume; ^b^*t*-Plot micropore volume; ^c^Mesopore volume obtained by subtraction of V_micro_ from V_total_; ^d^Mesopore diameter at the maximum of the pore size distribution curve.

**Figure 4:**
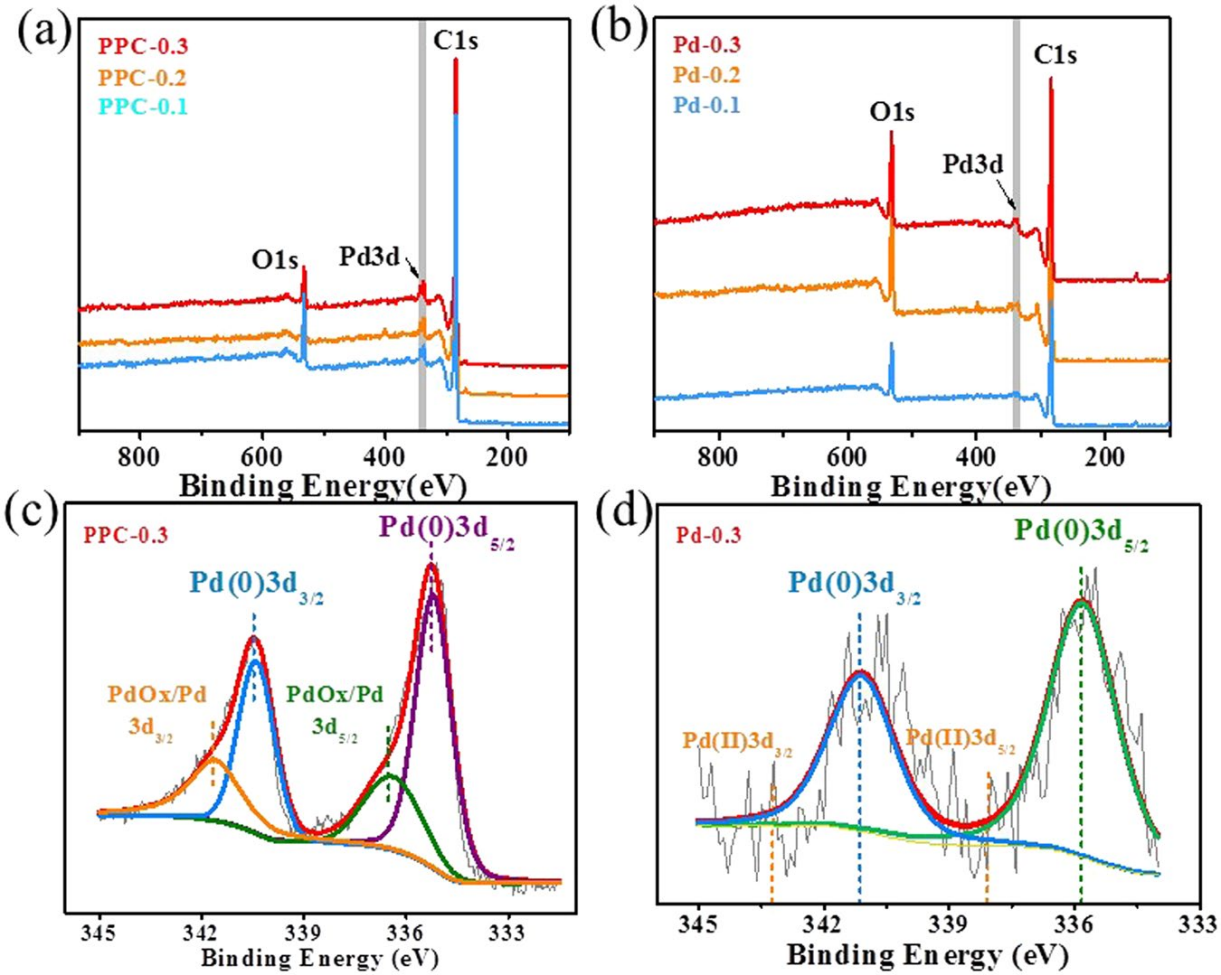
XPS spectra of (**a**) PPC samples with different Pd contents and (**b**) samples prepared without SiO_2_ with different Pd contents; High-resolution Pd3d XPS spectra of (**c**) PPC-0.3 and (**d**) Pd-0.3.

The XPS spectra in Fig. 4 show the elemental analysis of the PPC with different Pd contents and the samples prepared without SiO_2_ with different Pd contents, the binding energies were calibrated by C1s at 284.8 eV. It can be seen there were not only C and O signals ascribed to lignin in the samples, but also the signal of Pd due to the generation of Pd NPs. Compared with lignin-Pd NP composite, PPC samples after carbonization showed much stronger intensity of both C and Pd signals, which are agreement with XRD and ICPMS results. The high-resolution spectrums of Pd3d in Fig. 4c,d reveal the state of Pd element in detail. The peaks at 335.80 eV and 341.10 eV from Pd^0^ correspond to the Pd3d_5/2_ and Pd3d_3/2_, respectively^17^. In addition, a small proportion of Pd Ox/Pd mixtures at 336.40 eV and 341.70 eV are observed in Fig. 4c, which may be due to the interaction between Pd NPs and oxygen-contained groups within lignin during the carbonization process^18^. It is noted that the Pd3d spectrum of Pd-0.3 sample showed no peaks corresponding to Pd^2+^ at 338.00 eV and 343.30 eV, respectively, and indicated the negligible presence of Pd^2+^ species. The results confirmed the successful reduction of Pd NPs in one-pot synthesis, even though a small proportion of Pd Ox/Pd mixtures were introduced during carbonization process.

**Figure 5:**
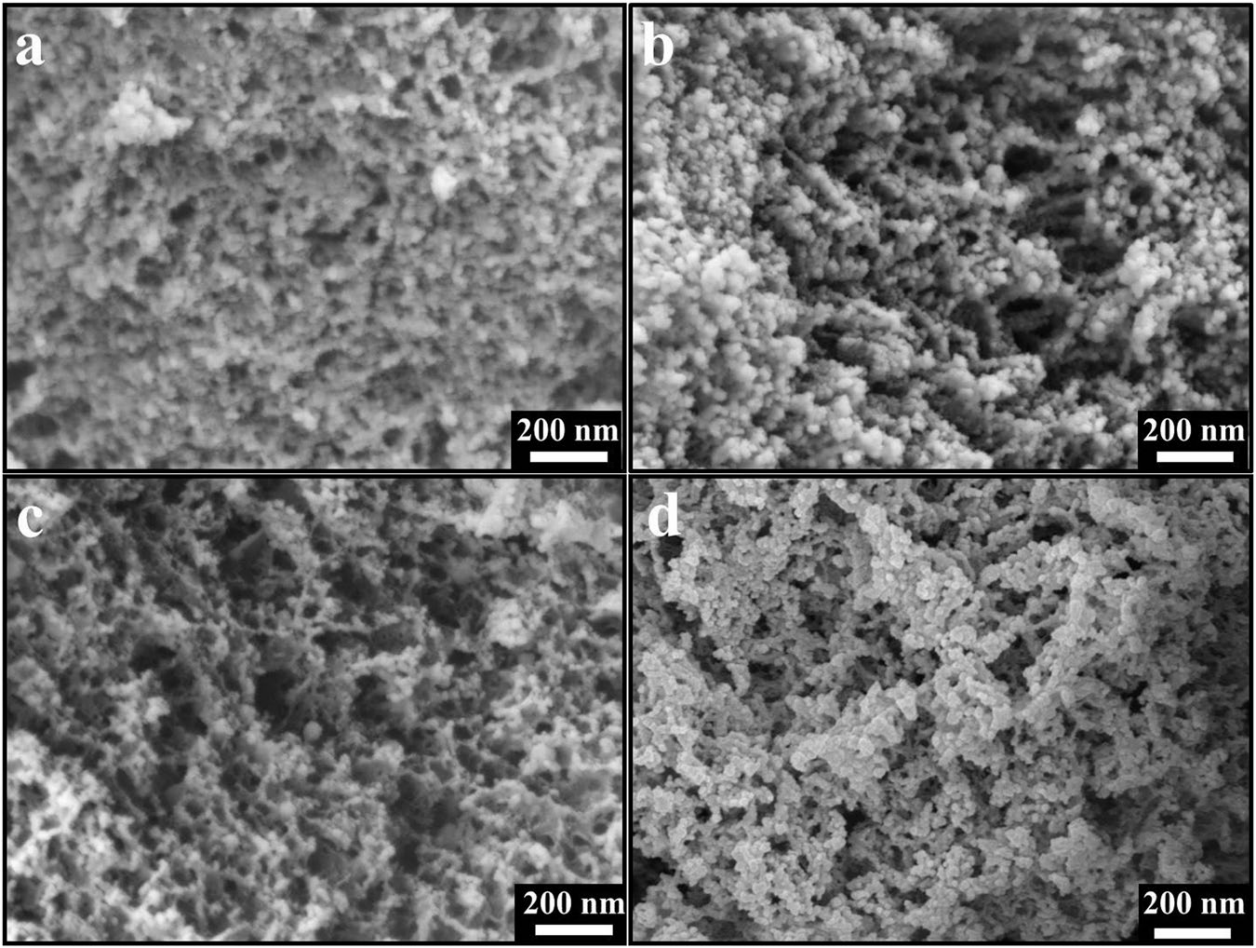
SEM images of (**a**) PPC-0.1, (**b**) PPC-0.2, (**c**) PPC-0.3, and (**d**) LPC.

Scanning electron micrographs are utilized to investigate the influence of Pd NPs on the texture properties of the resultant porous carbons. From the SEM images in Fig. 5(a–c), all the PPC samples exhibited excellent 3D porous structure, which are similar to the LPC sample shown in Fig. 5d. It is noteworthy that with the increasing Pd content, the porous structure of PPC sample seemed to be looser. It seems that when there were more Pd NPs dispersed in the network of lignin, there would be less interaction sites between SiO_2_ template and lignin molecules, so that looser structure would be obtained during carbonization process^13^.

**Figure 6:**
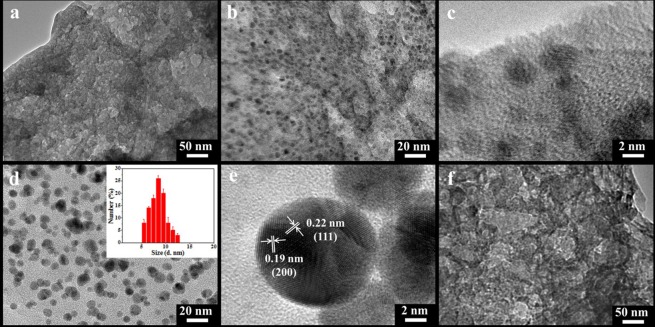
TEM images of (**a**–**c**) PPC-0.3 at different magnification, (**d**) Pd-0.3 (inset: the size distribution of Pd NPs), (**e**) one Pd nanoparticle from Pd-0.3, and (**f**) LPC.

Transmission electron microscope images in Fig. 6a show the porous structure of PPC-0.3 sample in large area, while the TEM images of LPC in Fig. 6f exhibit mesopores of 10 nm to dozens of nanometers in diameter, which are agreement with SEM results. In Fig. 6b, it can be observed that large amounts of small Pd NPs were homogeneously embedded in the carbon network and had good dispersity. And in the high-resolution TEM image (Fig. 6c), the spherical Pd NPs can be clearly seen, with similar shape and size to Pd NPs prepared with lignin as the reducing agent as shown in Fig. 6d,e, implying that carbonization process hardly influenced the shape and size of Pd NPs. The crystal detail of one Pd nanoparticle was further analyzed by high-resolution transmission electron microscopy (HRTEM). Figure 6e shows the grain boundaries, faults and dislocations on the surfaces of Pd nanoparticle. The measured d-spacing of 0.22 nm and 0.19 nm as shown in Fig. 6e corresponded to the (111) and (200) planes of the Pd crystals^19^, respectively, which is agreement with the XRD results.

### Properties of Pd nanoparticles-supported porous carbon (PPC) for hydrogen storage

**Figure 7:**
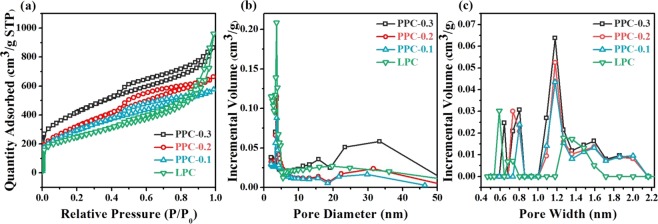
Textural Properties of the PPC and LPC samples: (**a**) nitrogen adsorption isotherms (**b**) mesopore size distribution curves and (**c**) micropore size distribution curves.

Lignin-based porous carbon samples without Pd NPs were prepared under different lignin/SiO_2_ ratios and carbonization temperatures, as given in Table S1. As shown in Figs S1, S2 and Table S2, it was found that the LPC samples with lignin/SiO_2_ ratios of 1:2 at 750 °C showed BET specific surface area of 893 m^2^/g, which is relatively high among lignin-based porous carbon materials without activation process^4,13,20^. Therefore, the Pd NPs-supported porous carbons were also prepared with lignin/SiO_2_ ratios of 1:2 at 750 °C. The structural properties of Pd NPs-supported porous carbons and LPC are evaluated by nitrogen adsorption-desorption isotherms, and the results are shown in Table 2 and Fig. 7. Nitrogen adsorption-desorption isotherms at 77 K (−196 °C) for PPC samples with different Pd contents are presented in Fig. 7a. As shown in Fig. 7a, the PPC samples had IV Type isotherms, according to the IUPAC nomenclature^21^, and had clearer hysteresis loops of H3 shapes than LPC sample, confirming the presence of mesopores. Moreover, the rapid adsorption in low relative pressure range (smaller than P/P_0_ = 0.1) also represented the presence of micropores^22^. Thus, the as-prepared PPC samples can be identified as micro-mesopore porous materials^23^. And with increasing residual Pd contents in final samples, the nitrogen adsorption of PPC samples increased, which is consistent with the results of BET specific surface area and total pore volume, as listed in Table 2. It is noteworthy that the BET specific surface area of PPC-0.3 was 1467 m^2^/g, which is much higher than that of LPC sample without supporting Pd NPs. This is because that high hydrogen spillover effects produced by residual Pd contents in samples greatly increased their specific surface area. And this indicated the improvement of texture properties by high Pd loading, which is agreement with the observed pore results of SEM and TEM. The mesopores size of PPC samples can be seen from the size distribution curves (Fig. 7b) by BJH method, as listed in Table 2. From the size distribution curves, two peaks were observed at around 15 and 30 nm, implying that there may be two types of mesopores structures within PPC samples. Moreover, with more Pd contents in lignin-Pd NPs composites, the mesopores size of resultant PPC sample was larger. The pore size distribution curves in the micropore range by the DFT method (Fig. 7c) indicate that under the same template dosage and carbonization temperature, higher Pd loading contents had positive impact on micropore volume (see Table 2).

**Table 2:** Hydrogen uptake of the PPC samples.

Samples	S_BET_ (m^2^/g)	H_2_ uptake condition	H_2_ uptake (wt. %)	H_2_ uptake condition	H_2_ uptake (wt. %)	Reference
PPC-0.1	1028	77 K/60 bar	1.06	298 K/60 bar	0.12	This study
PPC-0.2	1128	77 K/60 bar	1.49	298 K/60 bar	0.26	This study
PPC-0.3	1467	77 K/60 bar	3.52	298 K/60 bar	0.34	This study
LPC	893	77 K/60 bar	0.66	298 K/60 bar	0.08	This study
Pd-carbon nanotubes	30	77 K/68 bar	0.37	—	—	^27^
Co-modified carbon aerogels	970	77 K/40 bar	2.12	—	—	^26^
Nanostructured carbon-Pd	743	77 K/40 bar	2.83	298 K/40 bar	0.3	^28^
Pd/carbon nanotubes	—	—	—	298 K/ 68 bar	0.33	^29^
Pd/3D porous carbon	3316	77 K/60 bar	4.60	—	—	^13^
Activated carbon aerogels	3200	77 K/40 bar	5.31	—	—	^26^

The hydrogen storage capacities of the Pd nanoparticle-supported porous carbons were conducted and the results are shown in Table 2. It can be seen the H_2_ uptake capacity increases in the order of PPC-0.1 < PPC-0.2 < PPC-0.3, which means that with increasing residual Pd contents in final samples, the hydrogen storage capacity increased. These results can be ascribed to the process called spillover effect, which generally happens in such Pd doped samples^11,24^. When Pd NPs were dispersed in lignin-derived porous carbon, apart from physisorption of hydrogen in porous carbon, there was also chemisorption of hydrogen in Pd NPs, result in the formation of H atoms. The H atoms spill over towards the pores in PPC samples. Thus, higher Pd contents lead to stronger spillover effect, and enhanced hydrogen storage capacity^5,25^. Furthermore, it can be seen from Table 1 and Table 2 that PPC-0.3 sample with the highest micropore volume (0.18 cm^3^/g) exhibited much higher H_2_ uptake than the samples with lower micropore volume (0.06 cm^3^/g and 0.08 cm^3^/g). These results indicate that micropore volume of the porous carbons also has remarkable effect on the hydrogen storage capacity. When compared with some carbonaceous materials under similar condition (Table 2), the H_2_ uptake of prepared PPC samples was lower than Pd/3D porous carbon^9^, and activated carbon aerogels (with ultrahigh surface area)^26^. However, the PPC samples performed better than some metal-doped carbon materials such as Co-modified carbon aerogels^26^, Pd-carbon nanotubes^27^, nanostructured carbon-Pd^28^, Pd-decorated carbon nanotubes^29^, which was 3.52 wt. % under 77 K and 60 bar. And the maximum H_2_ uptake can reach to 3.98 wt. % under 77 K and 180 bar. As shown in Table 2, with the increase of H_2_ uptake temperature from 77 K to 298 K, H_2_ uptake capacity of all prepared samples decreased. In addition, the N_2_ adsorption isosteric technique was also applied to determine the isosteric heat with the calorimeter at 298 K for each sample as shown in Table 3. For N_2_, the isosteric heats decreased continuously with the increase of coverage. The parameter which may influence the H_2_ uptake is the surface chemistry. It can be explained that when the temperature is higher, higher internal energy and repulsive energy between hydrogen molecule make the adsorption reaction more difficult, and influence the spillover of hydrogen on prepared materials^28^. In this study, PPC samples were prepared without activation process after carbonization. It is believed that under optimized carbonization and activation process in upcoming study, the hydrogen storage capacity of PPC will be much better than now.

**Table 3:** Isosteric heats of adsorption of N_2_ on the PPC samples.

Samples	amount adsorbed (mol/kg)	isosteric heat (kJ/mol)
PPC-0.1	0.097	31.2
PPC-0.2	0.118	30.2
PPC-0.3	0.283	28.6
LPC	0.046	32.5

## Conclusions

A green strategy was applied for the synthesis of Pd NPs-supported porous carbon, utilizing lignin as not only reducing and stabilizing agents, but also the carbon source. The Pd NPs-supported porous carbon synthesized via hard-template method exhibited 3D interconnected mesoporous/macroporous carbon networks over a large area. The loading of Pd NPs was proved to increase the hydrogen storage capacity due to the spillover effects. Thus, the present study not only provides a promising hydrogen adsorbent based on lignin, more importantly, proposes a greener and more available strategy for efficient utilization of renewable materials.

## Experimental

### Materials and apparatus

Soda lignin, an abundant lignin species in Chinese paper industry as raw material, was obtained from the State Key Laboratory of Pulp & Paper Engineering (Guangzhou, China), recovering from wheat straw by the soda pulping process. Diammine dichloropalladium (II) (Pd(NH_3_)_2_Cl_2_) was purchased from Shanghai Macklin Biochemical Co., Ltd (Shanghai, China). Nano Silicon dioxide (99.5%, 15 ± 5 nm) was supplied by Shanghai Fortunebio-tech Ltd. (Shanghai, China). All other chemicals reagents and solvents are of analytical grade.

An XH-100A microwave synthesis system was purchased from Beijing XiangHu Sci.-Tech. Dept. Co., Ltd. (Beijing, China). The redox reaction is performed inside the system under pulsed microwave irradiation at atmospheric pressure.

### Synthesis of Pd nanoparticles-supported porous carbon (PPC)

Table 4 exhibits the preparation conditions and residual Pd contents of all carbon materials. For the preparation of Palladium nanoparticle-supported porous carbon, lignin/SiO_2_ mixture (1.5 g) with mass ratio of 1:2 was firstly dispersed in 1 wt. % NaOH solution. Pd(NH_3_)_2_Cl_2_ powder (0.1 mmol, 0.2 mmol, 0.3 mmol) was also dissolved in 20 mL 1 wt. % NaOH solution to obtain the Pd solution. The lignin/SiO_2_ dispersion was then added to the Pd solution, and the whole reactant was reacted under microwave irradiation (800 W) at 90 °C for 4 h. After lyophilization, the resultant samples were carbonized under 750 °C for 2 h under nitrogen flow (heating rate of 5 °C/min, flow rate = 60 cm^3^/min). After dissolving the SiO_2_ template in 10% HF at 80 °C for 6 h, the products were washed by deionized water and dried in the oven at 60 °C for 24 h. The resulting carbon materials were designated as PPC-p, where the p stands for different Pd dosages (mmol).

**Table 4:** Preparation conditions and residual Pd contents of all carbon materials.

Samples	Added Pd(NH_3_)_2_Cl_2_ powder (mmol)	SiO_2_ template	Carbonization process	Residual Pd contents (g/g)
PPC-0.3	0.3	with	with	0.6672
PPC-0.2	0.2	With	with	0.5560
PPC-0.1	0.1	with	with	0.4277
Pd-0.3	0.3	without	without	0.5920
Pd-0.2	0.2	without	without	0.4662
Pd-0.1	0.1	without	without	0.3986
LPC	without	with	without	—

For comparison, Pd NPs prepared with lignin as the reducing agent was conducted without SiO_2_ and prepared under the same microwave irradiation procedure, and the resultant black solution was dialyzed by ultrapure water and the solid product was obtained after lyophilization at −40 °C. The obtained products were denoted as Pd-0.1, Pd-0.2, Pd-0.3 respectively, corresponding to different Pd dosages. In addition, the lignin/SiO_2_ mixture was also treated without Pd NPs under the same recipe and procedure for comparison, and the as-prepared carbon was labeled as LPC.

### Characterization of Pd nanoparticles-supported porous carbon (PPC)

The UV-Vis spectrometry analysis was performed using a UV-1800 spectrophotometer (Shimadzu, Japan) with a scan range of 200~500 nm at a scan interval of 0.5 nm. Fourier transform infrared (FT-IR) analysis was performed using a Nicolet 5700 spectrophotometer (Madison, USA) under a dry air at room temperature by the KBr pellet method. The spectra were collected over the range of 4000 to 400 cm^−1^. X-ray diffractometer (XRD) analysis was performed on a D8 Advance XRD system (Cu Kα radiation, 40 kV, 50 Ma) (Bruker, Germany). X-ray photoelectron spectroscopy (XPS) analysis was performed using an AXIS Ultra DLD spectrometer (Kratos, UK) with Mg Kα radiation (hγ = 1253.6 eV) in step size of 0.1 eV. Transmission electron microscopy (TEM) images were obtained using a JEM-2100 (JEOL, Japan) instrument at an accelerating voltage of 200 kV. The residual Pd contents in final samples were detected using an Inductively Coupled Plasma Mass Spectrometer (ICPMS) (5300DV, Perkin Elmer, USA). Particle size analysis of Pd NPs was performed using a Malvern 3000HSA analyzer (Malvern, England). Pore textural analysis of lignin-based porous carbon was performed using an ASAP 2020 volumetric analyzer (Micromeritics, USA). The specific surface area was calculated using the Brunauer–Emmett–Teller (BET) method applied to adsorption data within the relative pressure (P/Po) range of 0.05–0.2. The total pore volume was calculated from the amount of nitrogen adsorbed at a relative pressure of 0.99, and the micropore volume was evaluated by t-plot method. The mesopore volume was obtained by subtraction of the micropore volume from the total pore volume. The pore size distribution (PSD) analysis was obtained via the Barrett-Joyner-Halenda (BJH) method from the desorption branch. In addition, the micro porosity of the porous carbon was estimated by the classical density functional theory (DFT) method using the nitrogen adsorption data.

## Supplementary information

Supporting information

## Data Availability

This manuscript comprises an original, unpublished material, which is not under consideration for publication elsewhere, and all authors have read and approved the text and consent to its publication. All experimental data are accurate and reliable.

## References

[1] Li X (2015). Dissolution of wheat straw with aqueous NaOH/Urea solution. Fibers & Polymers.

[2] Castro JB, Bonelli PR, Cerrella EG, Cukierman AL (2016). Phosphoric Acid Activation of Agricultural Residues and Bagasse from Sugar Cane:  Influence of the Experimental Conditions on Adsorption Characteristics of Activated Carbons. Industrial & Engineering Chemistry Research.

[3] Han G (2017). Special Magnetic Catalyst with Lignin-Reduced Au–Pd Nanoalloy. ACS Omega.

[4] Ju-Won J (2015). Controlling porosity in lignin-derived nanoporous carbon for supercapacitor applications. Chemsuschem.

[5] Hu S, Zhang S, Ning P, Hsieh YL (2014). High energy density supercapacitors from lignin derived submicron activated carbon fibers in aqueous electrolytes. Journal of Power Sources.

[6] Zhang W (2015). 3 D Hierarchical Porous Carbon for Supercapacitors Prepared from Lignin through a Facile Template-Free Method. Chemsuschem.

[7] Dipendu S (2014). Studies on supercapacitor electrode material from activated lignin-derived mesoporous carbon. Langmuir.

[8] Heggset EB, Syverud K, Øyaas K (2016). Novel pretreatment pathways for dissolution of lignocellulosic biomass based on ionic liquid and low temperature alkaline treatment. Biomass & Bioenergy.

[9] Coccia F, Tonucci L, Bosco D, Bressan M, D’Alessandro N (2012). One-pot synthesis of lignin-stabilised platinum and palladium nanoparticles and their catalytic behaviour in oxidation and reduction reactions. Green Chemistry.

[10] Modak A, Bhaumik A (2016). Surface-exposed Pd nanoparticles supported over nanoporous carbon hollow tubes as an efficient heterogeneous catalyst for the C C bond formation and hydrogenation reactions. Journal of Molecular Catalysis A Chemical.

[11] Zhu J (2014). One-pot synthesis of Pd nanoparticles on ultrahigh surface area 3D porous carbon as hydrogen storage materials. International Journal of Hydrogen Energy.

[12] Han G, Wang X, Hamel J, Zhu H, Sun R (2016). Lignin-AuNPs liquid marble for remotely-controllable detection of Pb2. Scientific Reports.

[13] Saha D, Payzant EA, Kumbhar AS, Naskar AK (2013). Sustainable mesoporous carbons as storage and controlled-delivery media for functional molecules. Acs Appl Mater Interfaces.

[14] Baca Martyna, Cendrowski Krzysztof, Banach Paweł, Michalkiewicz Beata, Mijowska Ewa, Kalenczuk Ryszard J., Zielinska Beata (2017). Effect of Pd loading on hydrogen storage properties of disordered mesoporous hollow carbon spheres. International Journal of Hydrogen Energy.

[15] Lu AH, Li WC, Hou Z, Schã¼Th F (2007). Molecular level dispersed Pd clusters in the carbon walls of ordered mesoporous carbon as a highly selective alcohol oxidation catalyst. Chemical Communications.

[16] Veerakumar P (2014). Highly stable and active palladium nanoparticles supported on porous carbon for practical catalytic applications^†^. Journal of Materials Chemistry A.

[17] Huriye EA, Onder M, Saim O (2009). *In situ*-generated PVP-stabilized palladium(0) nanocluster catalyst in hydrogen generation from the methanolysis of ammonia-borane. Physical Chemistry Chemical Physics.

[18] Moddeman WE, Bowling WC, Carter DC, Grove DR (2010). XPS Surface and Bulk Studies of Heat Treated Palladium in the Presence of Hydrogen at 150 °C. Surface & Interface Analysis.

[19] Zhang A (2013). Homogeneous Pd nanoparticles produced in direct reactions: Green synthesis, formation mechanism and catalysis properties. Journal of Materials Chemistry A.

[20] Fierro CM (2013). Colloidal templating synthesis and adsorption characteristics of microporous–mesoporous carbons from Kraft lignin. Carbon.

[21] Schiedt K, Leuenberger FJ, Vecchi M, Glinz E (1985). Absorption, retention and metabolic transformations of carotenoids in rainbow trout, salmon and chicken. Pure & Applied Chemistry.

[22] Gaikwad AV, Gadi R (2006). *In-situ* UV-visible study of Pd nanocluster formation in solution. Physical Chemistry Chemical Physics Pccp.

[23] Mayer ABR (1998). Formation of noble metal nanoparticles within a polymeric matrix: nanoparticle features and overall morphologies. Materials Science & Engineering C.

[24] Fernandes DM, Aaw H, Eag P (2006). Kinetic study of the thermal decomposition of poly(vinyl alcohol)/kraft lignin derivative blends. Thermochimica Acta.

[25] Das T, Banerjee S, Dasgupta K, Joshi JB, Sudarsan V (2015). Nature of Pd-CNT interaction in Pd nanoparticles dispersed multi-walled carbon nanotubes and its implications in Hydrogen storage properties. Rsc Advances.

[26] Kabbour H (2006). Toward New Candidates for Hydrogen Storage: High-Surface-Area Carbon Aerogels. Chemistry of Materials.

[27] Suttisawat Y (2009). Investigation of hydrogen storage capacity of multi-walled carbon nanotubes deposited with Pd or V. International Journal of Hydrogen Energy.

[28] Dibandjo (2013). Hydrogen storage in hybrid nanostructured carbon/palladium materials:;Influence of particle size and surface chemistry. International Journal of Hydrogen Energy.

[29] Jeng-Kuei C, Chih-Yao C, Wen-Ta T (2009). Decorating carbon nanotubes with nanoparticles using a facile redox displacement reaction and an evaluation of synergistic hydrogen storage performance. Nanotechnology.

