# Supplementary material for: Fabrication of Pd NPs-supported porous carbon by integrating the reducing reactivity and carbon-rich network of lignin
Source: Sci Rep. 2019 May 13;9:7300. doi: 10.1038/s41598-019-43840-0 (PMC6514013; doi:10.1038/s41598-019-43840-0)
Supplement: Supplementary file 1 — Supporting information [file 41598_2019_43840_MOESM1_ESM.docx]

**Fabrication of Pd NPs-supported porous carbon by integrating the reducing reactivity and carbon-rich network of lignin**

Guocheng Han^#^, Qimeng Jiang^#^, Weijie Ye, Chuanfu Liu, Xiaoying Wang^*^

*State Key Laboratory of Pulp & Paper Engineering, South China University of Technology, Guangzhou 510640, China*

^#^ Co-first author with the same contribution to this work

^*^ Corresponding author: Xiaoying Wang, E-mail: [xyw@scut.edu.cn](mailto:xyw@scut.edu.cn);

**Supporting information**

**Table S1** Lignin-porous carbons obtained from different reaction conditions

| **Samples** | **Lignin: SiO_2_ (mg:mg)** |  | **Carbonization temperature（°C）** |
| --- | --- | --- | --- |
| LPC0.5-500 | 1.0:0.5 |  | 500 |
| LPC1.0-500 | 1.0:1.0 |  | 500 |
| LPC2.0-500 | 1.0:2.0 |  | 500 |
| LPC0.5-750 | 1.0:0.5 |  | 750 |
| LPC1.0-750 | 1.0:1.0 |  | 750 |
| LPC2.0-750 | 1.0:2.0 |  | 750 |


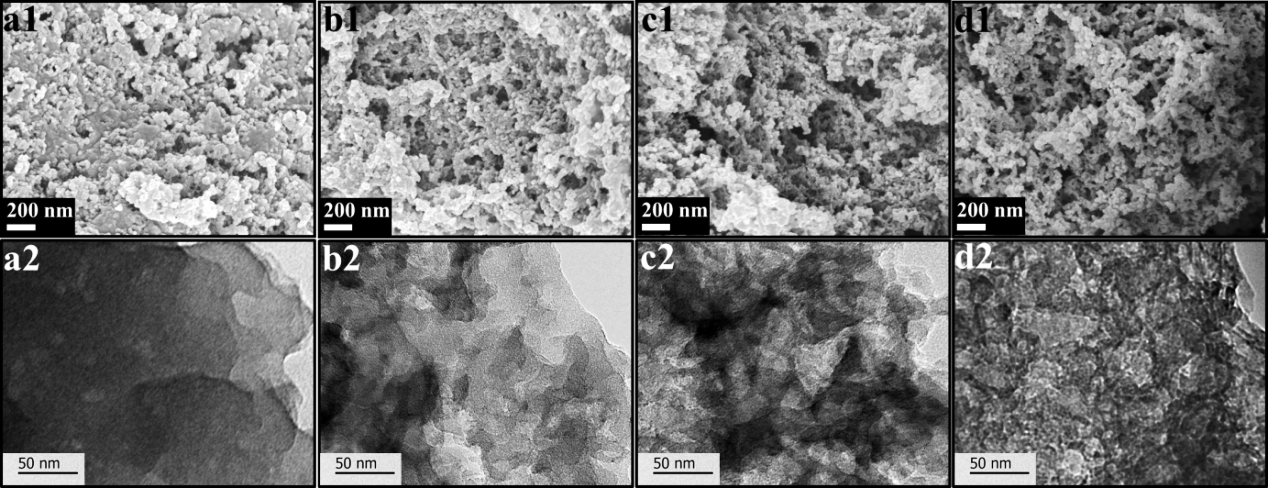


**Figure S1.** SEM images (1) and TEM images (2) of LPC samples: (a) LPC0.5-500, (b) LPC1.0-500, (c) LPC2.0-500 and (d) LPC2.0-750.


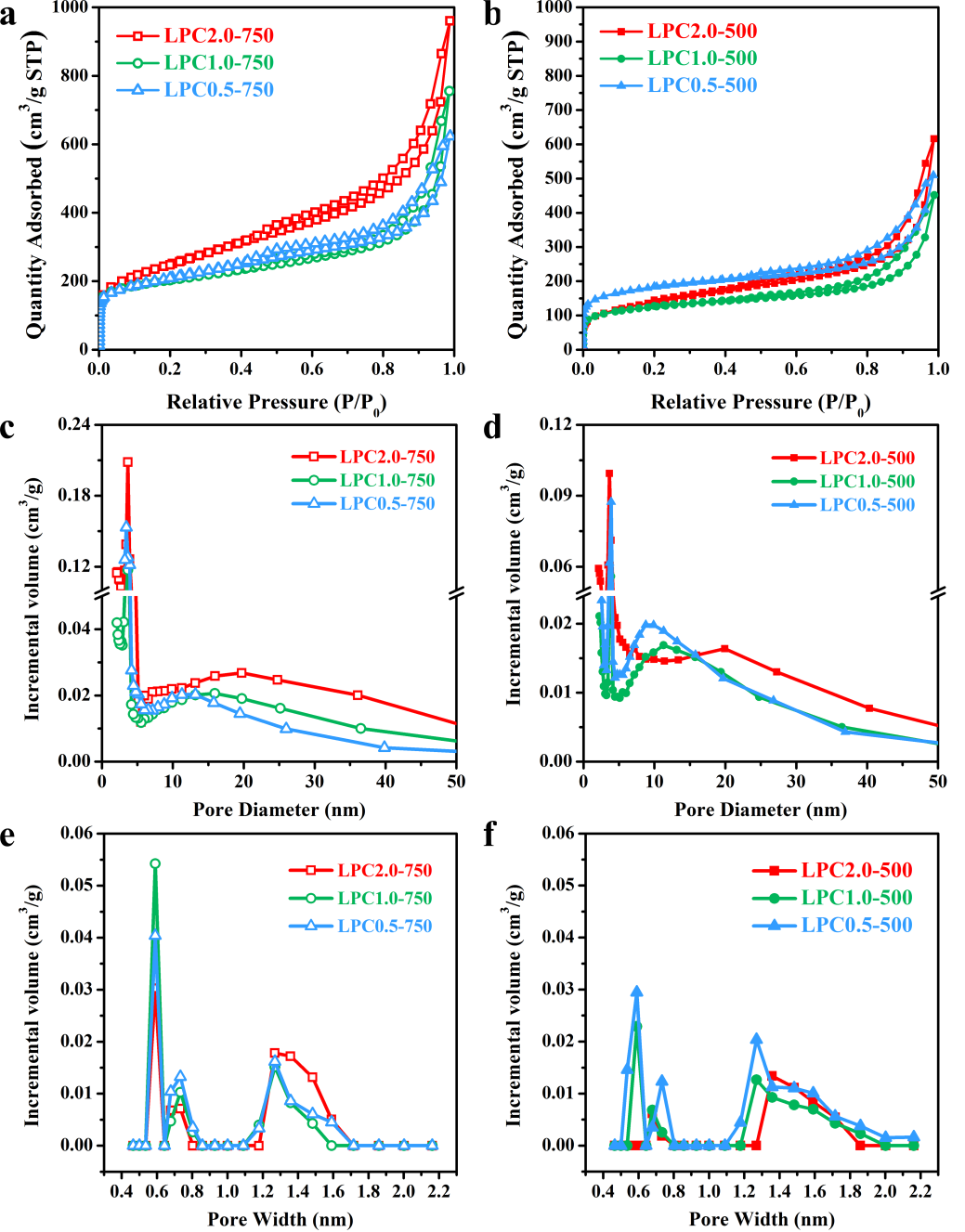


**Figure S2.** Nitrogen adsorption isotherms and the corresponding pore size distribution curves of LPC samples: (a), (c) and (e) samples carbonized at 750 °C; (b), (d) and (f) samples carbonized at 500 °C. The as-prepared carbons were labeled as LPCx-T, where *x* indicates the mass ratio of lignin to SiO_2_, and the *T* stands for the carbonization temperature.

**Table S2** Textural Properties of the LPC samples

| **Samples** | **S_BET_ (m^2^/g)** | **V_total_^a^ (cm^3^/g)** | **V_micro_^b^ (cm^3^/g)** | **V_meso_^c^ (cm^3^/g)** | **Pore size^d^ (nm)** |
| --- | --- | --- | --- | --- | --- |
| LPC0.5-500 | 635.10 | 0.79 | 0.10 | 0.69 | 8.78 |
| LPC1.0-500 | 436.71 | 0.70 | 0.08 | 0.62 | 11.30 |
| LPC2.0-500 | 502.21 | 0.95 | 0.02 | 0.93 | 19.92 |
| LPC0.5-750 | 730.59 | 0.96 | 0.11 | 0.85 | 11.28 |
| LPC1.0-750 | 704.35 | 1.17 | 0.15 | 1.02 | 15.92 |
| LPC2.0-750 | 892.69 | 1.49 | 0.05 | 1.44 | 19.73 |
| ^a^ total pore volume; ^b^ *t*-Plot micropore volume; ^c^ mesopore volume obtained by subtraction of V_micro_ from V_total_; ^d^ mesopore diameter at the maximum of the pore size distribution curve. | | | | | |
